# An international comparison of age and sex dependency of COVID-19 deaths in 2020: a descriptive analysis

**DOI:** 10.1038/s41598-021-97711-8

**Published:** 2021-09-27

**Authors:** Peter Bauer, Jonas Brugger, Franz König, Martin Posch

**Affiliations:** Section for Medical Statistics, Center for Medical Statistics, Informatics and Intelligent Systems, Medical University of Vienna, Spitalgasse 23, 1090 Vienna, Austria

**Keywords:** Diseases, Risk factors, Infectious diseases

## Abstract

The number of reported coronavirus disease (COVID-19) deaths per 100,000 persons observed so far in 2020 is described in 15 European countries and the USA as dependent on age groups and sex. It is compared with the corresponding historic all-cause mortality per year depending on age and sex observed in these countries. Some common features exist although substantial differences in age and sex dependency of COVID-19 mortality were noted between countries. An exponential increase with age is a good model to describe and analyze both COVID-19 and all-cause mortality above 40 years old, where almost all COVID-19 deaths occur. Moreover, age dependency is stronger for COVID-19 mortality than for all-cause mortality, and males have an excess risk compared with women, which is less pronounced in the higher age groups. Additionally, concerning calendar time, differences in the age and sex dependency between countries were noted with the common tendency that male excess risk for COVID-19 mortality was smaller in the second half of the year.

## Introduction

More than 1.7 million coronavirus disease (COVID-19) deaths have been recorded worldwide by December 27, 2020^1^. Age and sex were already observed as major risk factors for COVID-19 deaths early on in the pandemic, with a higher risk for males and an exponentially increasing risk with age^2,3^.

Case fatality rates can be considered, defined as the number of deceased over the number of detected infections, to investigate the risk patterns for COVID-19 mortality across countries. This number, however, does not account for the undetected infections and depends on the amount of testing and the testing strategy which substantially varied across time and between countries. By contrast, estimation of the infection fatality rate (IFR), defined as the ratio of deceased versus infected, relies on estimates of the actual number of infections, including the nondetected. Such estimates based on screening tests and seroprevalence studies have been provided for a range of countries^4,5^.

This paper focused on the analysis of the population mortality rate, defined as the ratio of deceased in a certain age and sex group and the population size in that group in a certain period. The latter quantity can be estimated based on COVID-19 mortality figures reported in different countries. However, the COVID-19 population mortality rates are an overall risk measure given by the product of the infection rate and the IFR in the respective subgroup.

The definitions and data sources of COVID-19 deaths are not standardized^6^ although information on the number is available for many countries^7^. Definitions on what constitutes a COVID-19 mortality range from any death within 28 days after a laboratory-confirmed COVID-19 infection to death for which a COVID-19 infection has been identified as the cause of mortality in the death certificate^7,8^. Analyses of excess mortality in several countries indicate that not all COVID-19 deaths were documented, although some of the excess mortality may also be due to indirect effects of the pandemic (e.g., overburdened health systems)^9^.

This study aims to descriptively analyze the age- and sex-specific COVID-19 population mortality rates in 15 European countries plus the USA for which sufficiently detailed numbers are available in the year 2020 from public sources. The present study especially investigates the age gradient and impact of sex on mortality and relates it to the corresponding patterns of all-cause mortality before the pandemic. Furthermore, time trends and a comparison of the mortality patterns in the first and second half of the year were assessed.

## Methods

Publicly available data of 14 countries on COVID-19 deaths by age and sex were taken from the National Institute for Demographic Studies (INED)^7^. Additionally, Spanish COVID-19 mortality data were acquired from the webpage of the Red Nacional de Vigilancia Epidemiológica^10^. Moreover, Austrian COVID-19 mortality data were requested from the Austrian National Public Health Institute (Gesundheit Österreich GmbH), which grants access to COVID-19 data for scientific research^11^. Furthermore, life table data of the most recent 5-year interval were used from the Human Mortality Database^12^. Data on the age- and sex-specific population sizes were taken either directly from the INED dataset or the latest information in the Human Mortality Database in the case of Austria and Spain (Supplementary Table 1S in the Supplementary Information for an overview of the datasets used).

Negative binomial regression models were fitted to the proportion of COVID-19 deaths in 2020 and to the proportion of all-cause deaths from the most recent life tables per country and year before 2020. Population fatality rates were analyzed and defined as the number of COVID-19 deaths divided by the total number of persons in the respective age groups. Separate analyses were conducted per country. In *Model 1 COVID*, the total numbers of COVID-19 deaths in 2020 were analyzed in a model with the metric variable age (centered at 65 years old and scaled in decades), the variable male sex (with female as the reference category), and the interaction age × sex with population size as an offset in each age by sex group. The same model was used to analyze all-cause mortality in the individual countries (*Model 1 ALL*). Although the age of death is available in years for all-cause mortality, the same age groups per country as in *Model 1 COVID* were used to avoid systematic differences arising from differences in the definition of the influence variables. Since all-cause mortality increases approximately exponentially with age from 40 years old and over (e.g., Refs.^13,14^), the models (*Model 1 All, Model 1 COVID*, and *Model 2 COVID*; see below) were fitted only in the subsamples of ≥ 40 years old, i.e., including only age groups that do not contain individuals below 40 years old. Overall, this covered 98.3% of the registered COVID-19 deaths for which information on age and sex was available in all countries. Sensitivity analysis calculations were repeated with an age limit of ≥ 30 years old, covering 99.1% of COVID-19 deaths, to check whether this age limit has an impact on the results. Since age was only available in age groups (except for Austria, where subject level data with age in years was available; see Supplementary Table 1S), it was approximated by the mean age of the population in the respective age group, accounting for age distribution. Because of the exponential increase of the proportions of deaths with age, this gives slightly positively biased estimates of the number of deaths at the mean age. In a sensitivity analysis for Austria, results using the 10-year age groups were compared with the results using the 1-year age groups. For countries where reported age groups varied over time, the age categorization applicable to all weeks was used.

For comparison of age and sex dependence of COVID-19 mortality between the first and second half of the year, a second model was fitted (*Model 2 COVID*). To this end, a dataset with weekly deaths was derived from the cumulative mortality figures, calculating the increments at the reported periods. Non-monotonicities over time due to temporal over-reporting were monotonized by setting negative increments to 0 and reducing the mortality counts in the immediately preceding period(s) by the respective amount (such that the total cumulative number of deaths per age and sex group remained unchanged). Weeks in which no deaths occurred were excluded from the dataset. Furthermore, the period was defined with period 1 indicating January 1, 2020, to June 31, 2020, and period 2 from July 1, 2020, to December 31, 2020, where the first period roughly corresponds to the first wave of the pandemic. *Model 2 COVID* included the independent-factor calendar week (as a categorical variable to account for the dynamics of total deaths over time), age, sex, two-way interactions (age × sex, age × period, and sex × period), and three-way interaction (age × sex × period). Period (with period 1 as the reference) was not included as main factor because it is completely confounded with the factor calendar week. Overdispersion was tested using the Cameron and Trivedi^15^ test. All analyses were conducted with R^16^, the negative binomial models were fitted with the glm.nb function in the MASS package^17^.

## Results

Definitions of COVID-19 deaths vary among countries, including clinically confirmed COVID-19 deaths, clinically confirmed COVID-19 deaths with laboratory confirmation of a SARS-COV2 infection, and all deaths within a certain period (e.g., 28 days) after a laboratory-confirmed infection. In some countries, the definitions, method of collection, and completeness changed over time. Table 1 gives an overview on the definitions and references to more detailed descriptions of the datasets. Supplementary Figure 1S (supplement) shows the cumulative total number of COVID-19 deaths for the 16 countries in the year 2020, together with the cumulative numbers for males and females in the age categories under and over 65 years (or under and over 60 years old, depending on the reported age categories)*.* Non-monotonicity of the cumulative numbers in individual age classes for women and men occurred in eight of the 16 countries and was removed as described in the “Methods” section for further analysis.

**Table 1:** Country overview and description of COVID-19 mortality definitions. Population size, number of COVID-19 deaths, the definition of COVID-19 deaths. If not indicated otherwise, the definition of COVID-19 deaths is taken from INED's metadata sheets^7^.

Country	Definition of COVID-19 deaths
Austria (AT)	A COVID-19 death is defined for surveillance purposes as a death resulting from a clinically compatible illness in a probable or confirmed COVID-19 case, unless there is a clear alternative cause of death that cannot be related to COVID disease (e.g., trauma). There should be no period of complete recovery between the illness and death. A death due to COVID-19 may not be attributed to another disease (e.g. cancer) and should be counted independently of pre-existing conditions that are suspected of triggering a severe course of COVID-19. Source: Direct inquiry at Gesundheit Österreich GmbH^11^
Belgium (BE)	Since 31 March 2020, both confirmed and suspected COVID-19 deaths have been included. Confirmation is based on a laboratory or radiological test. Source: https://www.ined.fr/fichier/rte/166/Page%20Data/Belgium/Metadata_Sheet_Belgium_v3.pdf
Switzerland (CHE)	Death of COVID-19 laboratory-confirmed cases Source: https://www.ined.fr/fichier/rte/166/Page%20Data/Switzerland/Metadata_Sheet_Switzerland_v1.pdf
Germany (DE)	Death of COVID-19 laboratory-confirmed cases. Source: https://www.ined.fr/fichier/rte/166/Page%20Data/Germany/Metadata_Sheet_Germany_v5.pdf
Denmark (DNK)	The cumulative number of deaths reported daily by the SSI corresponds to the number of deaths recorded within 30 days of detection of COVID-19 infection with a positive laboratory test. This definition was changed (from 60 to 30 days) on 29 March 2020. Source: https://www.ined.fr/fichier/rte/166/PageData/Denmark/Metadata_Sheet_Denmark_v5.pdf
England and Wales (ENW)	COVID-19 or suspected COVID-19 mentioned anywhere on the death certificate, including in combination with other health conditions. Certification is carried out according to the professional judgement of the attending physician based on available information such as symptoms and clinical findings, and does not require a positive virological test. Source: https://www.ined.fr/fichier/rte/166/Page%20Data/England%20and%20Wales/Metadata_Sheet_EnglandWales_v2.pdf
Spain (ESP)	Cases confirmed by PCR until May 10 2020, and by PCR and antigen testing from 11 May 2020 (according to Ref.^10^. In Ref.^7^ confirmation by IgM instead of antigen tests is stated). Sources^10^ and https://www.ined.fr/fichier/rte/166/Page%20Data/Spain/Metadata_Sheet_Spain_v2.pdf
France (FR)	Cumulative number of deaths of laboratory-confirmed SARS-CoV-2 (by RT-PCR or chest CT) that occurred in hospitals. Source: https://www.ined.fr/fichier/rte/166/Page%20Data/France/Metadata_Sheet_France_v5.pdf
Italy (IT)	COVID-19 tested deaths. COVID-19 laboratory confirmation is restricted to authorized laboratories since 19 May 2020. Source: https://www.ined.fr/fichier/rte/166/Page%20Data/Italy/Metadata_Sheet_Italy_v2.pdf
Netherlands (NLD)	Death of COVID-19 laboratory-confirmed cases. Source: https://www.ined.fr/fichier/rte/166/Page%20Data/Netherlands/Metadata-Sheet_TheNetherlands_v2.pdf
Norway (NOR)	Death of COVID-19 laboratory-confirmed cases. Source: https://www.ined.fr/fichier/rte/166/Page%20Data/Norway/Metadata_Sheet_Norway_v6.pdf
Portugal (PRT)	Death of COVID-19 laboratory-confirmed cases. Source: https://www.ined.fr/fichier/rte/166/Page%20Data/Portugal/Metadata_Sheet_Portugal_v2.pdf
Scotland (SCO)	Deaths where COVID-19 was mentioned on the death certificate, i.e. includes laboratory-confirmed, probable and suspected cases (cases where the doctor who certified the death noted that there was suspected or probable coronavirus infection involved in the death). Source: https://www.ined.fr/fichier/rte/166/Page%20Data/Scotland/Metadata_Sheet_Scotland_v3.pdf
Sweden (SWE)	Death of COVID-19 laboratory-confirmed cases. Source: https://www.ined.fr/fichier/rte/166/Page%20Data/Sweden/Metadata_Sheet_Sweden_v3.pdf
Ukraine (UKR)	Death of COVID-19 confirmed cases. Source: https://www.ined.fr/fichier/rte/166/Page%20Data/Ukraine/Metadata_Sheet_Ukraine_v3.pdf
United States of America (USA)	Confirmed (and, in many states suspected) COVID-19 deaths. The reports include laboratory-confirmed COVID-19 deaths and clinically-confirmed deaths where COVID-19 is listed as a “presumed” or “probable” cause (underlying or contributing), i.e. for which the certifier suspected COVID-19 was likely even without laboratory confirmation. As of October 1, 2020, COVID-19 is listed as the underlying cause on the death certificate in 92% of deaths. Source: https://www.ined.fr/fichier/rte/166/Page%20Data/USA/Metadata_Sheet_USA_v2.pdf

### Total numbers of COVID-19 deaths in 2020 (Model 1 COVID)

**Figure 1:**
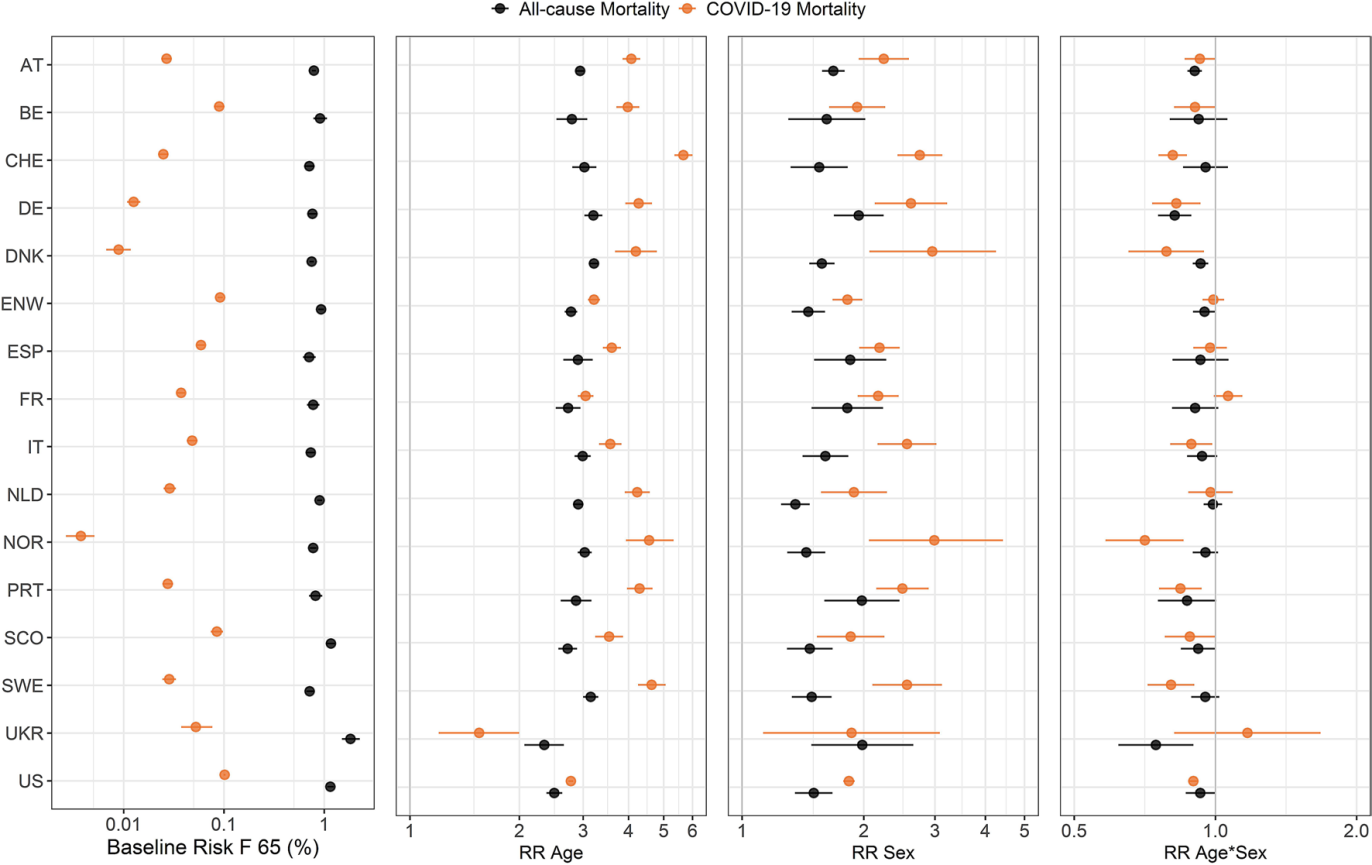
Analysis of all-cause and COVID-19 deaths depending on age and sex in 16 countries (Model 1). Estimates (dots) and 95% confidence intervals (lines) are obtained from the negative binomial model for COVID-19 deaths in 2020 (*Model 1 COVID*) and yearly all-cause mortality before 2020 (*Model 1 ALL*) for 16 countries. Given are the baseline risks for women at 65 years old (F 65), the risk ratios (RR) for age (per 10 years), for the male sex, and the interaction age × sex. Results for COVID-19 and all-cause deaths are in orange and black, respectively. Country codes are defined in Table 1.

**Figure 2:**
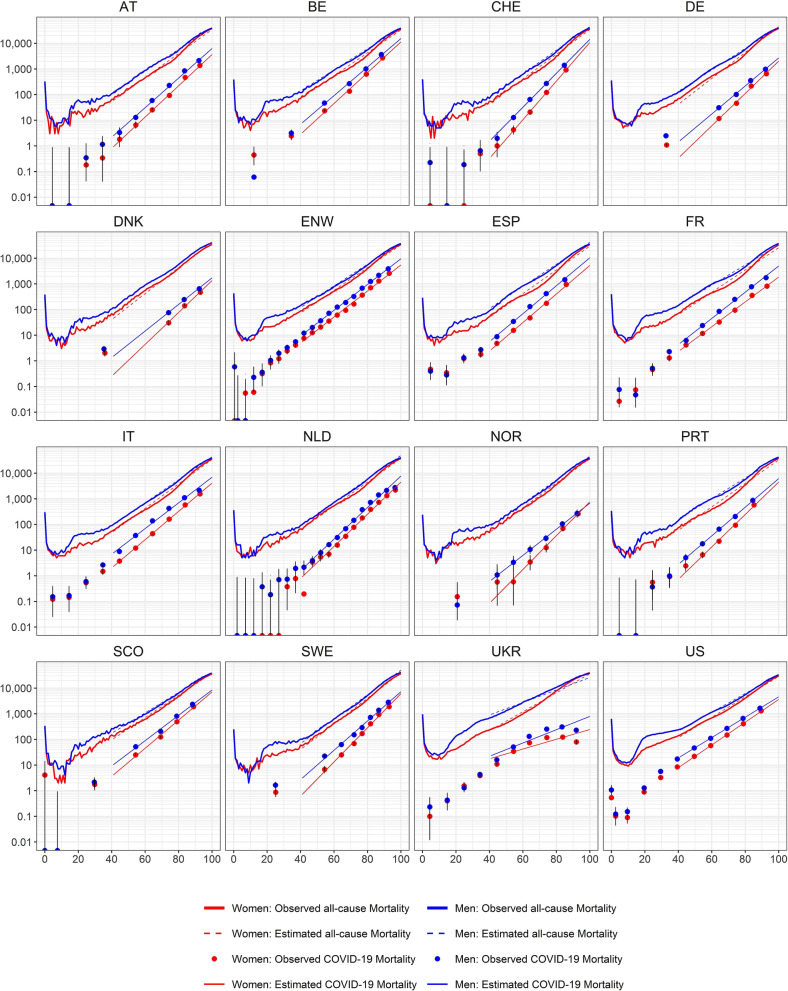
Age- and sex-specific all-cause and COVID-19 deaths in 16 countries. Predictions of Model 1 (see text) are given by straight lines on a log scale (dashed lines all-cause mortality, solid lines COVID-19 mortality). The curves show the number of persons who normally die per 100,000 within the next year depending on age and sex taken from all-cause mortality life tables per country before 2020 (red curves women; blue curves men). The dots are the number of COVID-19 deaths per 100,000 plotted at the means of age in the respective age categories. The vertical lines represent 95% confidence intervals with lower bounds cut at 0.001. The fitted model estimates for COVID-19 mortality in over 40 years old and all-cause mortality are shown by the solid and dashed lines from 40 years old and over. Country codes are defined in Table 1.

Figure 1 shows the estimated proportions of COVID-19 deaths for females at 65 years old (the estimated intercepts in the model) and the estimates of the risk ratios for age (per 10 years), sex, and the twofold interaction (age × sex) for COVID-19 mortality of persons over 40 years old in the year 2020 in the 16 countries. The corresponding risk ratios for the yearly all-cause mortality before 2020 estimated from the same model are shown in Fig. 1 for comparison. The numerical values per country (Supplementary Tables 2Sa,b) and the individual estimates of the risk ratios for the age dependency in females and males (Supplementary Fig. 2S) are presented in the Supplementary Information. Figure 2 presents the descriptive data and model fit for both types of mortality.

#### Mortality for 65-year-old females (reference group)

##### COVID-19 mortality

In the group of 65-year-old females (chosen as a reference), the lowest COVID-19 mortality risk was estimated for Norway, followed by Denmark and Germany with mortality rates of 0.004% (95% confidence interval, 0.003–0.005), 0.009% (0.007–0.012), and 0.013% (0.011–0.015), respectively. The highest risk was observed in the USA with 0.102% (0.099–0.104), with large values also in England and Wales (0.092%; 0.086–0.098), Belgium (0.090%; 0.080–0.101), and Scotland (0.085%; 0.074–0.098).

##### All-cause mortality

The estimated risk of all-cause deaths for women at 65 years old ranged from 0.71% (0.62–0.82) in Spain and 0.72% (0.66–0.78) in Sweden up to a maximum of 1.83% (1.50–2.27) in Ukraine, followed by Scotland with 1.17% (1.07–1.28; see Supplementary Table 2Sb).

#### Age dependency

##### COVID-19 mortality

The smallest risk ratio for age (per 10 years) for COVID-19 mortality was observed in Ukraine with 1.55 (1.20–2.00; Fig. 1 and Supplementary Table 2Sa). This means that the risk of COVID-19 mortality in women (the reference category for the variable sex) increased by a factor of 1.55 times if age increased by 10 years. The next smallest age dependency was found in the USA with a risk ratio of 2.77 (2.73–2.82). A remarkably wide range of the estimates of age dependency was noted with the largest risk ratio observed in Switzerland (5.66; 5.36–6.00). The risk ratios for Belgium (3.98; 3.70–4.28), and Austria (4.07; 3.85–4.31) were closest to the median of the 16 countries.

##### All-cause mortality

The risk ratios for age varied from 2.34 (2.07–2.66) to 3.21 (3.13–3.30) for Ukraine (lowest) and Denmark (highest), respectively. The risk ratios for Spain (2.90; 2.65–3.19) and the Netherlands (2.91; 2.82–3.00) were closest to the median among the countries.

#### Sex dependency

##### COVID-19 mortality

COVID-19 mortality for males at 65 years old was consistently larger than for females. It was closest to females in England and Wales with a risk ratio of only 1.82 (1.68–1.98) for males versus females. The estimated risk ratio was largest in Norway at 2.99, although with a wide confidence interval (2.06–4.42). This is a remarkable spread of estimates of the countries. The estimated risk ratios of 2.19 (1.95–2.45) for Spain and 2.24 (1.95–2.59) for Austria were closest to the median of the 16 countries.

##### All-cause mortality

The estimated risk ratios for all-cause mortality between men and women at 65 years old were consistently above 1, but lower than the COVID-19 mortality (except for Ukraine). They ranged from 1.35 (1.25–1.47) in the Netherlands to 1.98 (1.48–2.65) in Ukraine. However, Portugal and Germany were also close to this high value (Fig. 1 and Supplementary Table 2Sb). The risk ratios for Denmark (1.58; 1.47–1.69) and Italy (1.61; 1.41–1.83) were closest to the median in the countries.

#### Interaction term for sex and age

##### COVID-19 mortality

Estimates of the interaction between age and sex suggest that the difference between male and female mortality rates was smaller for higher age groups. The risk ratio of males versus females over a decade of age reduced most pronouncedly (by a factor of 0.71; 0.58–0.86) in Norway. For 10 of the 16 countries, the 95% confidence intervals for the risk ratio corresponding to the interaction age × sex were completely below 1; the six other confidence intervals covered the value of 1 (Fig. 1 and Supplementary Table 2Sa).

##### All-cause mortality

Similarly, the observed difference between sexes is smaller for higher age, and the point estimates of the interactions between age and sex were below 1 for all countries. The confidence intervals exclude 1 for five of the 16 countries (Fig. 1 and Supplementary Table 2Sb).

#### Sensitivity analyses for Model 1 COVID and Model 1 all

In a sensitivity analysis, 30 (instead of 40) years was applied as a lower limit for age. As a result, the corresponding estimates for the interaction age × sex for all-cause mortality slightly shifted toward smaller values. Moreover, the corresponding confidence intervals fell below 1 (data not shown) in nine countries (compared with five countries with the 40-year threshold). Other effect estimates for COVID-19 and all-cause mortality were not noticeably affected by the lower age threshold. The estimated age effect for COVID-19 mortality was larger in this sensitivity analysis only for Ukraine. This is due to a subexponential increase of risk with age in Ukraine. The latter also caused a poorer model fit and mostly wider confidence intervals (see Fig. 2).

In a further sensitivity analysis with Austrian data, the present study investigated if the results change if the models are fitted with 1-year age intervals (available in Austria) compared with those with 10 years (available for many other countries). No relevant deviations between the estimates arising from the differences in length of the age categories were found (see Supplementary Table 2Sa).

### Correlation of age dependency of COVID-19 and all-cause mortality

**Figure 3:**
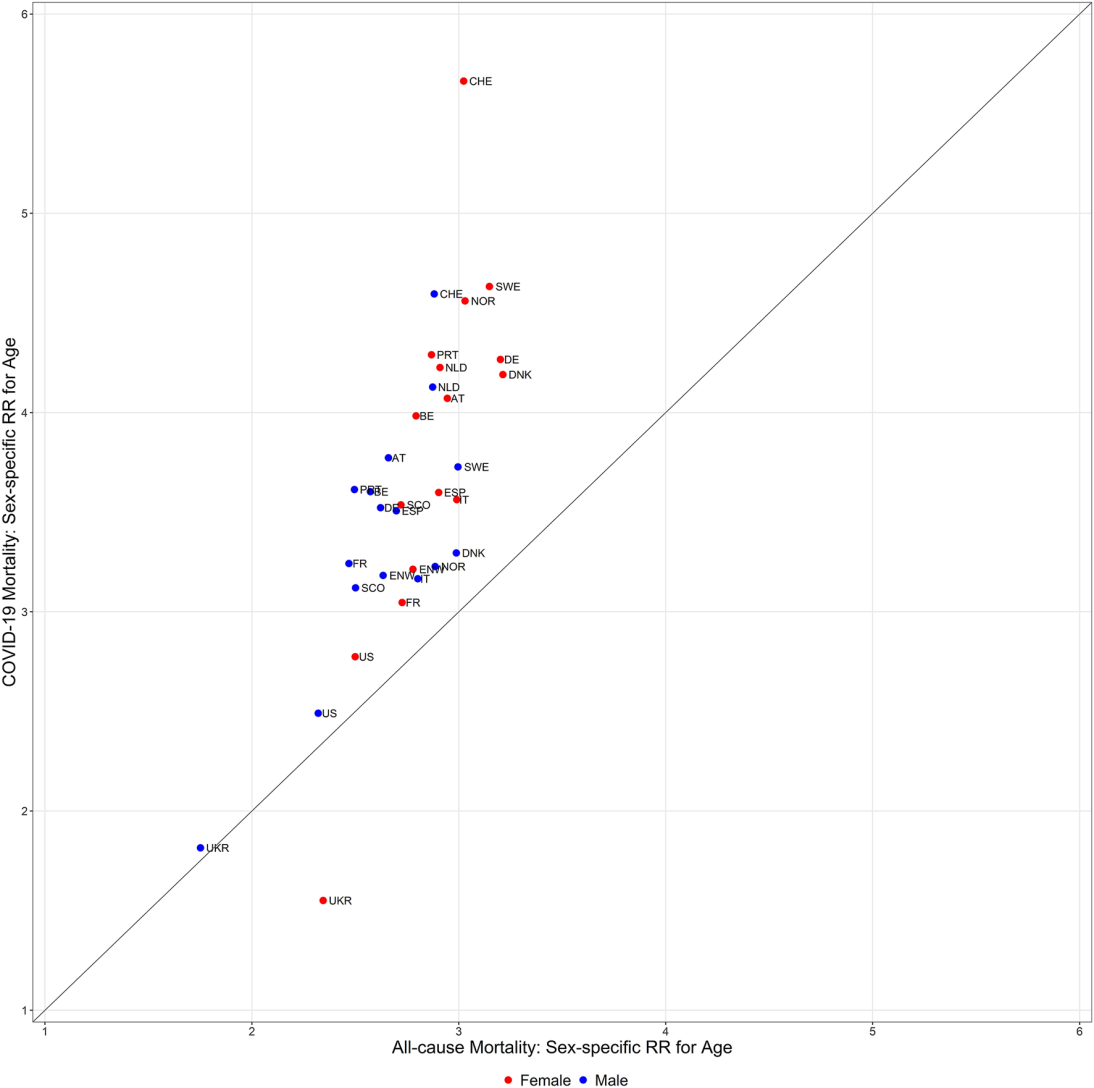
Association of the risk ratios for age between all-cause and COVID-19 deaths. The scatterplot shows the risk ratios for the age of males (blue dots) and females (red dots) estimated for COVID-19 mortality in 2020 versus the corresponding risk ratios estimated for all-cause mortality before 2020. The risk ratios are estimated from *Model 1 COVID* and *Model 1 ALL*. Country codes are defined in Table 1.

Figure 3 shows a scatterplot of the estimates of the risk ratios for age dependency in males and females of COVID-19 mortality versus the corresponding estimates for all-cause mortality per country and year, computed from *Model 1 COVID and Model 1 ALL*. Overall, a considerable correlation was noted between the two estimated risk ratios (Spearman Rho for males and females together *r* = 0.72, *n* = 32) despite the existing heterogeneity of results. When looking separately at males (Spearman Rho 0.50, *n* = 16) and females (Spearman Rho *r* = 0.79, *n* = 16), this suggests a possibly less stringent correlation of the estimates for males.

### Comparison of the age and sex dependency of COVID-19 mortality between periods (Model 2 COVID)

**Figure 4:**
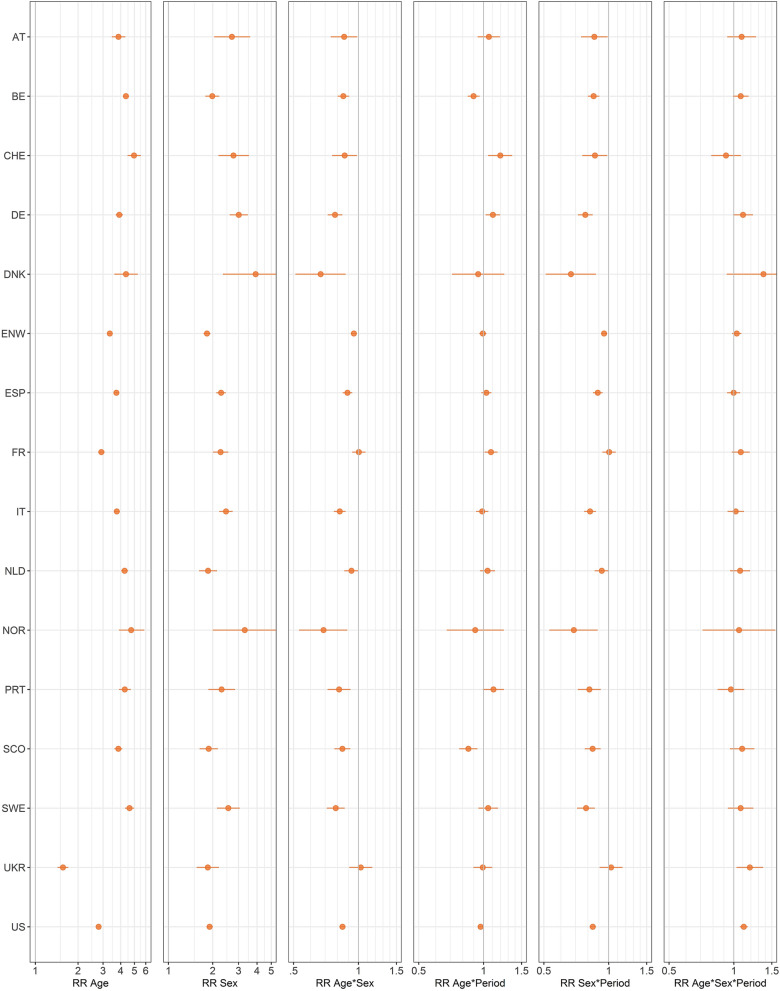
Analysis of COVID-19 mortality in dependency of age, sex, and period in the 16 countries. Estimates and 95% confidence intervals for the risk ratios of age (per 10 years), sex, period, twofold interactions (age × sex, age × period, sex × period), and the threefold interaction (age × sex × period) for COVID-19 mortality from *Model* 2 COVID. Country codes are defined in Table 1.

Figure 4 and Supplementary Table 3S show the estimates of the main effects of age and sex as well as the interactions of age × period, sex × period, and age × sex × period from Model 2 COVID.

#### Change in age dependency between periods

Changes in age dependency of COVID-19 mortality between the first and second half of the year 2020 varied across countries. The age dependency for women in Belgium, Scotland, and the USA was less accentuated in the second half of the year with point estimates and confidence intervals for the risk ratio of the interaction age × period falling below 1. The corresponding point estimates and confidence intervals were above 1 in Switzerland, Denmark, and France. Supplementary Figure 3S shows the individual estimates of the sex-specific risk ratio for the age per period, showing a lower age dependency of COVID-19 mortality for men compared with that for women in both periods, but with substantial variability between countries.

#### Change in sex dependency for 65 years old between periods

The only consistent effect across the 16 countries was the smaller sex difference in COVID-19 mortality for 65 years old in the second compared with that in the first half of the year. The confidence intervals of the corresponding risk ratios for the interaction period × sex are completely below 1 in 13 countries (Fig. 4) and cover 1 in the remaining countries. Because the risk ratios for the main effect of sex are all above 1, this implies that the difference between 65-year-old men and women decreased in the second period.

#### Change of the interaction age × sex between periods

Point estimates for the threefold interaction (age × sex × period) above 1 were noted in 13 of the 16 countries. However, only two of them had a confidence interval of above 1. A comparison of the age × sex interactions in periods 1 and 2 showed in 12 countries an attenuation of the deviation of the age dependencies between males and females in the second period of 2020 (Supplementary Fig. 4S).

## Discussion

This study aims to describe the age and sex dependencies of COVID-19 deaths as documented in 15 European countries and the USA. Overall, the fit of the models appears to be good, and the fit for all-cause mortality above 40 years old is hardly distinguishable from the life table data (Fig. 2). Hence, the exponential growth of the risk of death with increasing age is ascertained to be a good model for describing and analyzing both COVID-19 and all-cause deaths over 40 years old. The reported numbers show a deviating behavior, and COVID-19 mortality appears to be not monotonically increasing with age, leading to a poor model fit, only in Ukraine. While the overall model fits appeared to be good, age, sex and calendar week alone cannot individually predict death from COVID-19 in 2020^18^ and also in high risk groups the observed proportion of people that died due to COVID-19 was low.

Overdispersion has been tested using the Cameron and Trivedi test and found a noticeable overdispersion (*p* < 0.05) in *Model 1 COVID* (*Model 2 COVID*) in nine (12) of the 16 countries. Therefore, throughout neg-binomial models were used.

For many countries, the low numbers of registered COVID-19 deaths at young ages make it difficult to infer any convincing results for these age groups as may be seen from the wide confidence intervals in Fig. 2. However, the patterns of COVID-19 and all-cause deaths appeared to be similar even in these low ages for large countries (e.g., the USA). Higher risk in the first year of life was observed, which decreased for older children but then increased with age for young adults.

Considerable differences are noted in the estimated age and sex dependency of COVID-19 mortality between countries, and several confidence intervals of the risk ratios for age and sex do not overlap between countries. However, the percentage of increase in COVID-19 mortality per 10 years of increasing age was generally larger than the percentage of increase of all-cause mortality over 10 years of age in the countries before the pandemic (Supplementary Fig. 2S). Roughly, in terms of the median over countries, the risk of COVID-19 mortality in 2020 for an increase in age of 10 years increased by a factor of around 4, whereas it only increased by a factor of slightly below 3 for all-cause mortality. Comorbidities, which are more prevalent in older age groups, are risk factors for both COVID-19 and all-cause mortality^19,20^. A potential explanation for the more pronounced age dependency of COVID-19 mortality could be that comorbidities have a larger impact on COVID-19 than for other causes of mortality. Another reason for the higher COVID-19 age dependency could be the clusters of infections in nursing homes^21^ where the prevalence of comorbidities is high^22^.

It is not surprising that estimates of the risk ratios for age correlate between COVID-19 and all-cause deaths over countries because both types of mortalities are higher in subjects with comorbidities^20^. A correlation between COVID-19 and all-cause mortality has been reported also in Ref.^23^ where sex was not considered as a factor.

Also, the excess of COVID-19 mortality in men compared with that in women in terms of risk ratios was more accentuated for COVID-19 mortality than for all-cause mortality. Moreover, the median risk ratio for COVID-19 deaths in males compared with that in females at 65 years old was about 2.2 compared with 1.6 for all-cause mortality. In the majority of the countries, the age dependency of the excess male risk of COVID-19 mortality decreased with age. This trend is known also for all-cause mortality, and no indication was found that the magnitude of the trend would differ much between COVID-19 and all-cause deaths in different countries. Whereas the male-to-female ratio of the absolute numbers of COVID-19 deaths was substantially above 1/2 for the lower age groups, it was more balanced for higher age groups (Supplementary Fig. 1S). Thus, one may expect larger interaction effects between age and sex at first glance. However, the numbers at risk are larger for females than for males in higher age groups.

From early data in China, South Korea, Italy, Spain, France, Germany, England and Wales, and the USA up to April 2020 without differentiation for sex, all-cause mortality was found to be increasing exponentially at a constant rate of about 10% per year of age while COVID-19 mortality increased at about 11% per year of age^23^. This is similar to the estimates of the present study for yearly increases of around 12% and 15% corresponding to 10 years risk ratios of 3 and 4, respectively. In an analysis of data from eight European countries up to May 2020 differentiating sex, the progression ratios per 10 years of age of COVID-19 mortality has been estimated as 3.3 and 3.4 for females and males, respectively^14^, which does not indicate any narrowing of the excess risk of men with increasing age. Ahrenfeldt et al.^24^ for 10 European countries reported crude relative risks for males in four age groups from data collected up to June 29, 2020. They found the smallest differences between males and females in the age group over 85 years old.

To investigate time trends in *Model 2 COVID*, in addition to the variables already used in *Model 1*, the calendar week and interactions of the period (the second versus the first half of the year) with age and sex up to the threefold interaction were included. The main factor for the period cannot be estimated in a model including the calendar weeks. However, *Model 2* allows investigating whether changes in age and sex dependencies of COVID-19 mortality existed during 2020. In this analysis, differences in age dependency of COVID-19 mortality between the first and second half of the year were found among the 16 countries. For some countries, age dependency was more accentuated in the second half of the year and was more attenuated in the first half for others. This is consistent with the analysis of data in 14 countries in Ref.^25^, where the age distribution of COVID-19 deaths (however, pooling males and females and focusing on the proportion of deaths under 50 years of age) was compared between the two waves in 2020 and only small fluctuations were reported.

In the current analysis that distinguished between males and females, a consistent decrease of the excess mortality of males in the second half of the year was observed. To explain this effect, one would have to know whether some common systematic differences exist between females and males over countries in contacts, behavior, and infections over the pandemic and if or why such differences may have leveled out during the pandemic.

Although consistent in the qualitative trends, the age and sex dependency of COVID-19 mortality varied quite noticeably between countries. Moreover, per country analyses were conducted to obtain parsimonious models. Conservative between-country comparisons of effects can still be made based on the reported confidence intervals (nonoverlapping of confidence intervals asymptotically imply significant differences at a two-sided significance level of 0.05)^26^. However, observed differences between countries can be affected by the heterogeneity of definitions of COVID-19 deaths and differences in data collection (see the paragraph on limitations below).

Villani^27^ concluded that countries within Europe have performed very differently in their responses to the COVID-19 pandemic, but limitations in the available data must be addressed before a definitive assessment of the reasons for these differences can be made. Similarly, identification of causes for sex- and age-specific differences in COVID-19 mortality between specific countries is limited by the available data and the many possible sources for the observed variability as, e.g., differences in the measures against the pandemic, in the willingness of the populations to adhere to advice, in testing, in the health system, in the health awareness of the populations, in elderly care, in the registration procedures of COVID-19 deaths, in the political situation, and so on. Additionally, these factors may have differently impacted the different age and sex groups^23,25,28–32^. Interpretations of these differences require detailed information on specific events occurring and measures taken during the pandemic within each country (or regions within the country), but also considering geographic location^28^. For the interpretation, one has to keep in mind that differences in all-cause mortality between countries also exist. However, demographic characteristics between countries have been successfully used to explain differences in mortality between countries^29,30,33^ and factors as lower income or lower education have been identified as risks^31,32^.

To summarize, the key findings of this work are (1) an exponential increase of COVID-19-related mortality exists with age, (2) a higher age dependency for COVID-19-related mortality compared with all-cause mortality (especially in women), (3) mitigation of sex-specific differences of COVID-19 mortality in higher age groups, and (4) the excess risk of COVID-19 mortality in men compared with that in women decreased in the second half of the year.

Our study has several limitations. The country comparison of COVID-19 mortality may be confounded by different definitions of COVID-19 deaths as well as different coverage^8^. Based on^7^ and additional sources, we describe the definitions of death due to COVID-19 used (Table 1). However, for several countries only limited information has been published^6^. Similarly, changes in the COVID-19 mortality may be confounded with changes in the definition of COVID-19 deaths for the comparison between periods. As this analysis focuses on the association of age and sex with COVID-19 mortality, the heterogeneity in the definitions of COVID-19 deaths may have an impact on the findings if it differently affects the considered age cohorts and sexes.

The analysis is restricted to population fatality rates of COVID-19 and does not allow direct conclusions on the corresponding IFRs. The population fatality rates are substantially lower than the IFRs if only a small proportion of the population is infected. This may confuse public perception if not appropriately communicated. The population fatality rates depend on infection rates and IFRs. This implies that age and sex dependencies found for the population fatality rate cannot be directly extrapolated to corresponding dependencies for the IFRs unless a constant infection rate over age groups and sex per country applies.

As in most countries, the definition of COVID-19 deaths depends on laboratory-confirmed infections, and the reported numbers potentially can be confounded with the amount and method of COVID-19 testing of patients that died. Overall testing was much more prevalent in the second half of the year. However, this may only bias the results if the testing of COVID-19 patients that died differs systematically between age cohorts and sexes. A further limitation is that the periodically published cumulative mortality figures in some countries are not monotonic in time for some reporting periods (Supplementary Fig. 1S). This does not affect Model 1 (which has no period effect). Moreover, the non-monotonicities (if occurring) for Model 2 are mostly small and have been adjusted for in the analysis (see “Methods” section). The adjustment is expected to reduce potential bias but cannot fully correct for the over-reporting (as no information in which periods the over-reporting occurred was available).

## Supplementary Information

Supplementary Information.

## Data Availability

With the exception of the Austrian data, all datasets used in this analysis are publicly available (see Supplementary Table 1S in the Supplementary Information for the data sources). The Austrian data has been provided from the Austrian National Public Health Institute^11^ which provides access to the data set for scientific research institutions.
