# Supplementary material for: An international comparison of age and sex dependency of COVID-19 deaths in 2020: a descriptive analysis
Source: Sci Rep. 2021 Sep 27;11:19143. doi: 10.1038/s41598-021-97711-8 (PMC8476584; doi:10.1038/s41598-021-97711-8)
Supplement: Supplementary file 1 — Supplementary Information. [file 41598_2021_97711_MOESM1_ESM.pdf]

Supplementary Material for “An international comparison of age and sex dependency of COVID-19 deaths in 2020—a descriptive analysis”

Peter Bauer, Jonas Brugger, Franz König, Martin Posch

Section for Medical Statistics, Center for Medical Statistics, Informatics and Intelligent Systems, Medical University of Vienna, Spitalgasse 23, 1090 Vienna, Austria

Country	Population size	COVID-19 deaths	COVID-19 deaths with missing age or sex	Age categories	Average number of reports per week	Weeks without report	Dataset
AT	8,859,650	5,306	0.00	1-year interval	7.00	–	Data set provided by Gesundheit Österreich GmbH https://datenplattform-covid.goeg.at/
BE	11,492,641	19,163	0.19	0–24, 25–44, 45–64, 65–74, 75–84, 85+	6.93	–	Deaths_by_occurrence
CHE	8,582,905	6,777	0.02	0–9, 10–19, 20–29, 30–39, 40–49, 50–59, 60–69, 70–79, 80+	3.18	32, 33, 36, 37	Dataset name missing (see table caption)
DE	83,019,213	26,964	0.16	0–59, 60–69, 70–79, 80–89, 90+	4.62	38	SSI_by age and sex_Data
DNK	5,822,763	1,070	11.13	<70, 70–79, 80–89, 90+	5.30	–	Daily Report RKI_Data
ENW	59,115,809	73,730	0.00	<1, 1–4, 5–9, 10–14, 15–19, 20–24, 25–29, 30–34, 35–39, 40–44, 45–49, 50–54, 55–59, 60–64, 65–69, 70–74, 75–79, 80–84, 85–89, 90+	1.00	–	ONS_WeeklyOccurrenceDeaths
ESP	46,937,063	50,610	0.01	0–9, 10–19, 20–29, 30–39, 40–49, 50–59, 60–69, 70–79, 80+	7.00	–	Data obtained from RENAVE [10]
FR	67,063,703	42,615	1.06	0–9, 10–19, 20–29, 30–39, 40–49, 50–59, 60-69, 70–79, 80–89, 90+	6.78	–	SpF_by age and sex_HospitalData
IT	60,359,546	67,537	<0.01	0–9, 10–19, 20–29, 30–39, 40–49, 50–59, 60–69, 70–79, 80–89, 90+	1.36	–	Combined_Information
NLD	17,282,163	10,633	0.00	0–4, 5–9, 10–14, 15–19, 20–24, 25–29, 30–34, 35–39, 40–44, 45–49, 50–54, 55–59, 60–64, 65–69, 70–74, 75–79, 80–84, 85–89, 90–94, 95+	2.90	–	RIVM_Data
NOR	5,367,580	429	0.00	<40, 40–49, 50–59, 60–69, 70–79, 80–89, 90+	4.68	–	FHI_Deaths by age and sex
PRT	10,276,617	6,674	0.05	0–9, 10–19, 20–29, 30–39, 40–49, 50–59, 60–69, 70–79, 80+	6.89	34–47	min-sau_Data
SCO	5,463,300	6,298	0.00	0, 1–14, 15–44, 45–64, 65–74, 75–84, 85+	1.00	–	NRS Age & Sex
SWE	10,327,553	7,802	0.00	0–49, 50–59, 60–69, 70–74, 75–79, 80–84, 85–89, 90+	1.12	–	NHBW_Data
UKR	41,732,779	16,446	0.00	0–9, 10–19, 20–29, 30–39, 40–49, 50–59, 60–69, 70–79, 80–89, 90+	6.60	–	Deaths by age and sex
USA	327,167,434	291,749	<0.01	0, 1–4, 15–24, 25–34, 35–44, 45–54, 5–14, 55–64, 65–74, 75–84, 85+	1.00	–	CDC_Data

Table 1S. Overall population, COVID-19 deaths in 2020, percentage of deaths reported for which no information on age and/or sex was available, age groups for which deaths were reported, the average number of reports per weeks during the reporting period, weeks in which no updated figures are available, and data source. Except for Austria and Spain, the data source refers to the name of the datasheet obtained from INED [7] (see the *Methods* section in the main manuscript). Data from Switzerland were obtained from INED, but Switzerland is not listed in their metadata table.

a. COVID-19 mortality in 2020

Country	Baseline risk F 65 (%)	RR age	RR sex	RR age × sex
AT	0.027 (0.024–0.030)	**4.07 (3.85–4.31)**	**2.24 (1.95–2.59)**	**0.93 (0.86–1.00)**
	0.028 (0.024–0.032)	***4.12 (3.83–4.45)***	***2.18 (1.83–2.61)***	*0.94 (0.85–1.04)*
BE	0.090 (0.080–0.101)	**3.98 (3.70–4.28)**	**1.93 (1.64–2.26)**	0.90 (0.82–1.00]
CHE	0.025 (0.022–0.028)	**5.66 (5.36–6.00)**	**2.75 (2.43–3.13)**	**0.81 (0.76–0.87)**
DE	0.013 (0.011–0.015)	**4.27 (3.92–4.64)**	**2.62 (2.13–3.22)**	**0.83 (0.73–0.93)**
DNK	0.009 (0.007–0.012)	**4.19 (3.67–4.78)**	**2.96 (2.07–4.25)**	**0.79 (0.65–0.95)**
ENW	0.092 (0.086–0.098)	**3.21 (3.10–3.34)**	**1.82 (1.68–1.98)**	0.99 (0.94–1.04)
ESP	0.059 (0.054–0.064)	**3.60 (3.40–3.81)**	**2.19 (1.95–2.45)**	0.97 (0.90–1.06)
FR	0.037 (0.034–0.041)	**3.05 (2.90–3.20)**	**2.17 (1.93–2.44)**	1.06 (0.99–1.14)
IT	0.048 (0.043–0.055)	**3.56 (3.32–3.83)**	**2.56 (2.17–3.03)**	**0.89 (0.80–0.98)**
NLD	0.029 (0.025–0.033)	**4.23 (3.91–4.58)**	**1.89 (1.57–2.28)**	0.98 (0.88–1.09)
NOR	0.004 (0.003–0.005)	**4.56 (3.94–5.32)**	**2.99 (2.06–4.42)**	**0.71 (0.58–0.86)**
PRT	0.028 (0.024–0.031)	**4.29 (3.96–4.66)**	**2.49 (2.15–2.89)**	**0.84 (0.76–0.93)**
SCO	0.085 (0.074–0.098)	**3.54 (3.24–3.86)**	**1.86 (1.53–2.25)**	**0.88 (0.78–1.00)**
SWE	0.028 (0.024–0.033)	**4.63 (4.25–5.06)**	**2.56 (2.10–3.12)**	**0.80 (0.72–0.90)**
UKR	0.052 (0.038–0.077)	**1.55 (1.20–2.00)**	**1.87 (1.13–3.08)**	1.17 (0.82–1.68)
USA	0.102 (0.099–0.104)	**2.77 (2.73–2.82)**	**1.84 (1.78–1.90)**	**0.90 (0.88–0.92)**

b. All-cause mortality

Country	Baseline risk F 65 in %	RR age	RR sex	RR age × sex
AT	0.789 (0.754–0.827)	**2.94 (2.87–3.02)**	**1.68 (1.58–1.79)**	**0.90 (0.87–0.94)**
BE	0.909 (0.782–1.066)	**2.79 (2.53–3.08)**	**1.62 (1.30–2.02)**	0.92 (0.80–1.06)
CHE	0.711 (0.635–0.800)	**3.02 (2.80–3.26)**	**1.55 (1.32–1.83)**	0.95 (0.85–1.06)
DE	0.764 (0.692–0.846)	**3.20 (3.02–3.39)**	**1.94 (1.69–2.24)**	**0.82 (0.75–0.89)**
DNK	0.750 (0.713–0.789)	**3.21 (3.13–3.30)**	**1.58 (1.47–1.69)**	**0.93 (0.90–0.97)**
ENW	0.931 (0.871–0.996)	**2.78 (2.67–2.89)**	**1.46 (1.33–1.60)**	0.95 (0.90–1.00]
ESP	0.709 (0.615–0.822)	**2.90 (2.65–3.19)**	**1.85 (1.51–2.27)**	0.93 (0.81–1.07)
FR	0.776 (0.675–0.899)	**2.73 (2.52–2.95)**	**1.82 (1.49–2.23)**	0.91 (0.81–1.01)
IT	0.736 (0.672–0.808)	**2.99 (2.84–3.15)**	**1.61 (1.41–1.83)**	0.94 (0.87–1.01)
NLD	0.901 (0.851–0.955)	**2.91 (2.82–3.00)**	**1.35 (1.25–1.47)**	0.99 (0.94–1.03)
NOR	0.776 (0.718–0.839)	**3.03 (2.90–3.17)**	**1.44 (1.29–1.61)**	0.95 (0.90–1.01)
PRT	0.820 (0.708–0.958)	**2.87 (2.60–3.16)**	**1.98 (1.60–2.45)**	0.87 (0.75–1.00]
SCO	1.168 (1.067–1.282)	**2.72 (2.56–2.89)**	**1.47 (1.29–1.68)**	0.92 (0.84–1.00]
SWE	0.718 (0.663–0.778)	**3.15 (3.00–3.30)**	**1.49 (1.33–1.67)**	0.95 (0.89–1.02)
UKR	1.832 (1.503–2.266)	**2.34 (2.07–2.66)**	**1.98 (1.48–2.65)**	**0.75 (0.62–0.90)**
USA	1.155 (1.072–1.247)	**2.50 (2.38–2.63)**	**1.50 (1.35–1.67)**	**0.93 (0.86–1.00)**

Table 2S. Estimates and 95% confidence interval from the negative binomial model for COVID-19 deaths in 2020 (*Model 1 COVID*; Table a) and all-cause mortality (*Model 1 ALL*; Table b). Baseline risk (in percentage) for women at 65 years old, risk ratio (RR) for age per 10 years, male sex, and the interaction of age × sex in 16 countries. Boldface indicates that the RR significantly differs from 1. For confidence intervals with the rounded boundary of 1.00, square and round brackets indicate that 1 is included and excluded, respectively. The second row for Austria (in italics) shows the estimate of the model fitted with age categorized in 10-year age intervals.

Change “10-year age intervals”
to “1-year age intervals “

Country	RR age	RR sex	RR age × sex	RR age × period	RR sex × period	RR age × sex × period
AT	**3.86 (3.46–4.31)**	**2.70 (2.05–3.60)**	**0.86 (0.74–0.99)**	1.06 (0.94–1.19)	**0.86 (0.74–0.99)**	1.09 (0.93–1.27)
BE	**4.35 (4.16–4.56)**	**1.99 (1.78–2.22)**	**0.85 (0.80–0.91)**	**0.90 (0.84–0.96)**	**0.85 (0.80–0.91)**	1.08 (0.99–1.17)
CHE	**4.97 (4.47–5.55)**	**2.77 (2.19–3.53)**	**0.86 (0.75–0.99)**	**1.20 (1.05–1.36)**	**0.86 (0.75–0.99)**	0.92 (0.79–1.08)
DE	**3.91 (3.69–4.14)**	**3.01 (2.61–3.47)**	**0.78 (0.72–0.84)**	**1.11 (1.02–1.19)**	**0.78 (0.72–0.84)**	1.10 (0.99–1.23)
DNK	**4.36 (3.61–5.28)**	**3.92 (2.35–6.61)**	**0.67 (0.51–0.87)**	0.94 (0.71–1.25)	**0.67 (0.51–0.87)**	1.37 (0.93–2.04)
ENW	**3.35 (3.27–3.44)**	**1.83 (1.73–1.93)**	**0.95 (0.92–0.98)**	0.99 (0.96–1.03)	**0.95 (0.92–0.98)**	1.03 (0.98–1.08)
ESP	**3.73 (3.59–3.88)**	**2.28 (2.12–2.46)**	**0.89 (0.85–0.94)**	1.03 (0.98–1.09)	**0.89 (0.85–0.94)**	1.00 (0.93–1.07)
FR	**2.92 (2.78–3.08)**	**2.26 (2.01–2.56)**	1.00 (0.94–1.08)	**1.08 (1.01–1.16)**	1.00 (0.94–1.08)	1.08 (0.98–1.18)
IT	**3.75 (3.58–3.94)**	**2.46 (2.22–2.74)**	**0.82 (0.77–0.87)**	0.99 (0.92–1.05)	**0.82 (0.77–0.87)**	1.02 (0.94–1.11)
NLD	**4.26 (4.03–4.51)**	**1.86 (1.62–2.14)**	0.93 (0.86–1.00]	1.04 (0.96–1.13)	0.93 (0.86–1.00)	1.07 (0.96–1.19)
NOR	**4.74 (3.89–5.87)**	**3.31 (2.00–5.66)**	**0.69 (0.53–0.89)**	0.91 (0.68–1.24)	**0.69 (0.53–0.89)**	1.06 (0.72–1.56)
PRT	**4.27 (3.89–4.71)**	**2.30 (1.87–2.84)**	**0.81 (0.72–0.92)**	1.11 (0.99–1.24)	**0.81 (0.72–0.92)**	0.97 (0.84–1.12)
SCO	**3.85 (3.62–4.11)**	**1.88 (1.63–2.17)**	**0.84 (0.77–0.92)**	**0.85 (0.77–0.94)**	**0.84 (0.77–0.92)**	1.09 (0.96–1.24)
SWE	**4.61 (4.29–4.97)**	**2.55 (2.14–3.06)**	**0.78 (0.71–0.86)**	1.05 (0.95–1.17)	**0.78 (0.71–0.86)**	1.08 (0.94–1.23)
UKR	**1.56 (1.43–1.71)**	**1.85 (1.56–2.21)**	1.03 (0.91–1.16)	0.99 (0.90–1.10)	1.03 (0.91–1.16)	**1.19 (1.03–1.37)**
US	**2.79 (2.73–2.86)**	**1.91 (1.81–2.00)**	**0.84 (0.82–0.87)**	**0.97 (0.94–1.00)**	**0.84 (0.82–0.87)**	**1.11 (1.07–1.16)**

Table 3S. Estimates and 95% confidence interval from the negative binomial model for COVID-19 mortality in 2020 (*Model 2 COVID*) based on weekly mortality counts. RR for age per 10 years, male sex, and the interactions of age × sex, age × period, sex × period, and age × period × period in 16 countries. Estimates for the factor calendar week are not shown. Boldface indicates that the RR is significantly different from 1. For confidence intervals with the rounded boundary of 1.00, square and round brackets indicate that 1 is included and excluded, respectively.

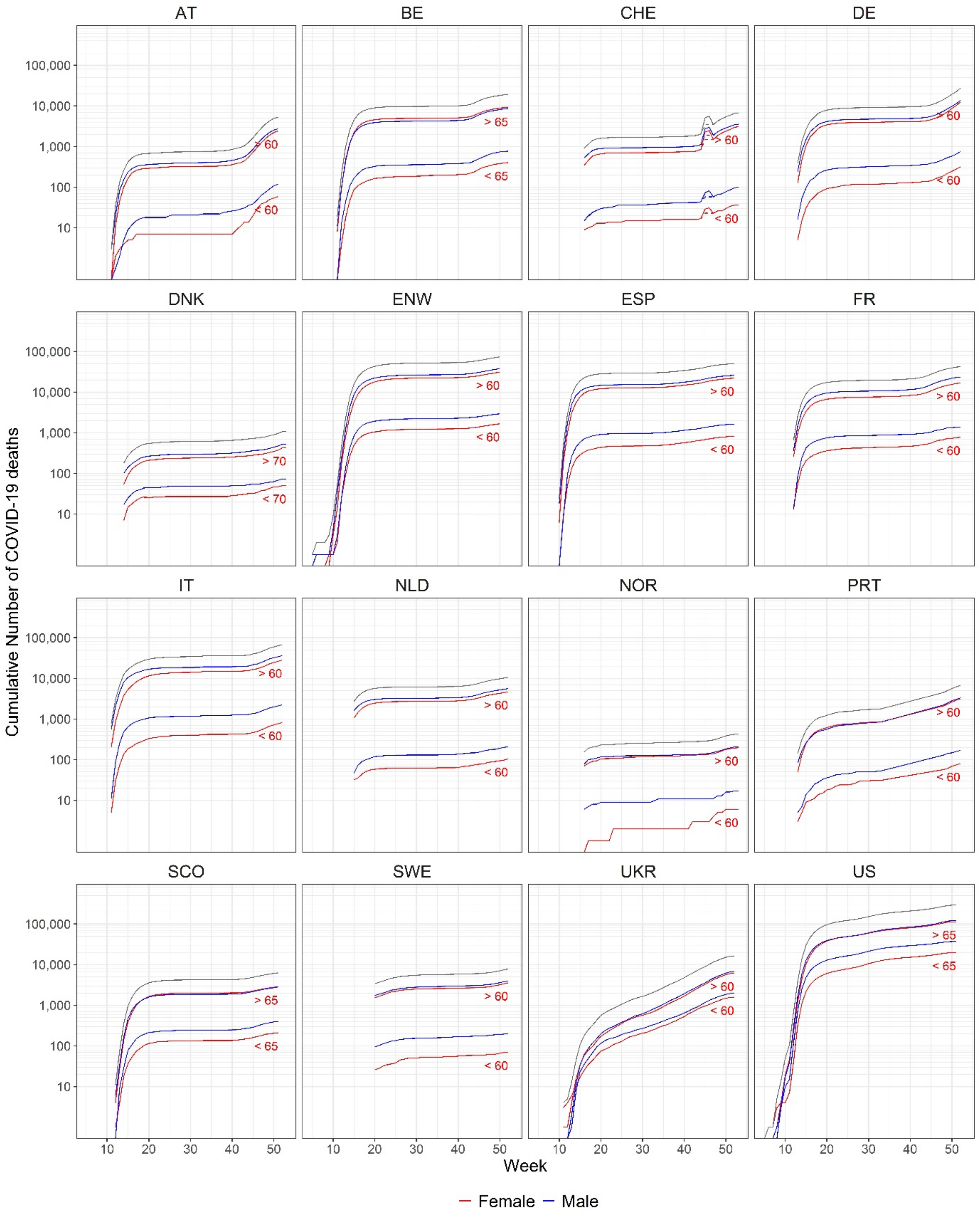

Figure 1S. Logarithm of the cumulative numbers of COVID-19 deaths over the calendar weeks of 2020 in 16 countries. *Black* total numbers, *red* female, *blue* male both for deaths ≤60 (or ≤65) and >60 (or >65) years old. For some countries (e.g., Switzerland), data errors seem to exist and the monotonized by age group (see the *Methods* section in the main manuscript) are given by *dashed lines*.

Change "monotonized by age group" to "monotonized values by age group"

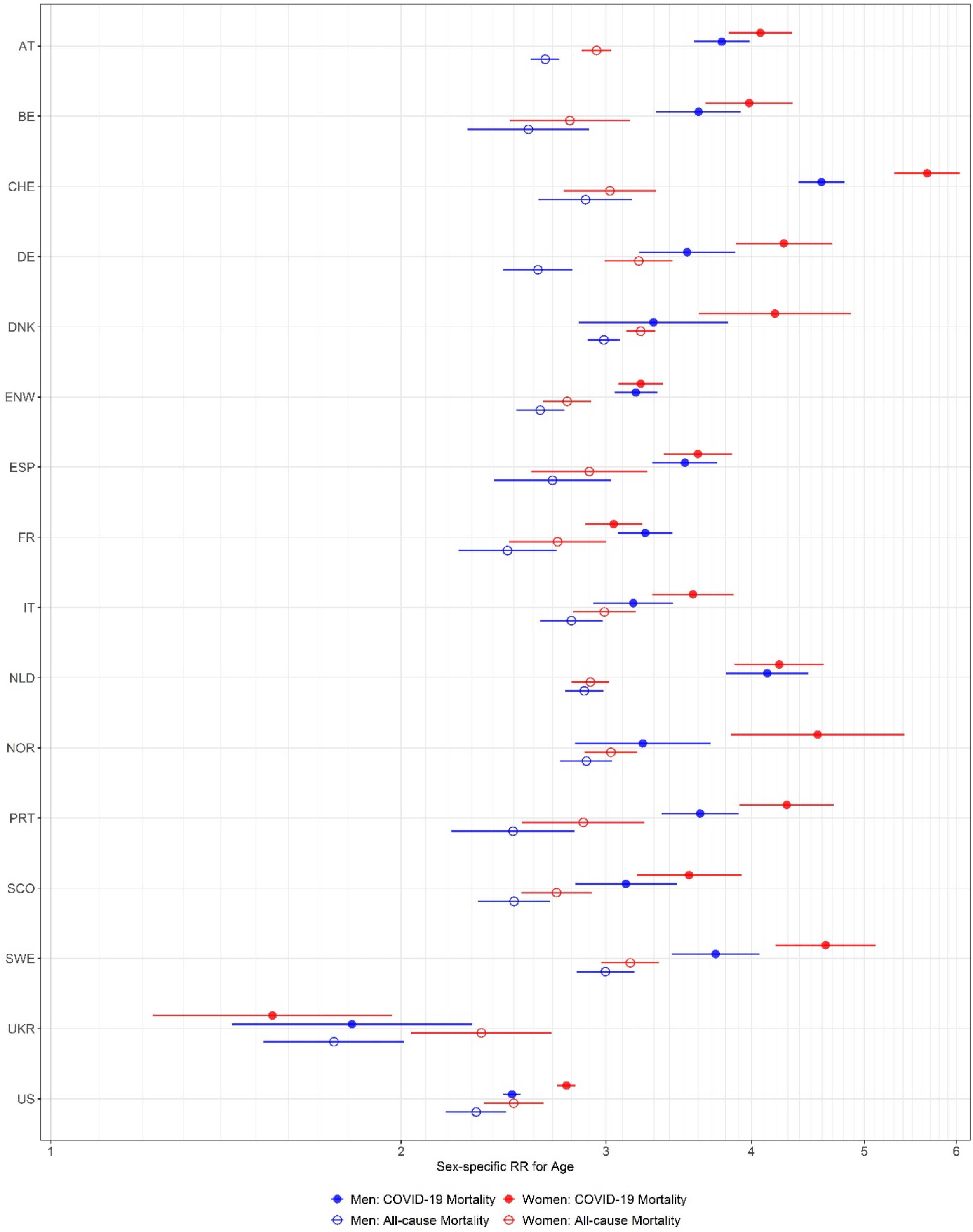

Figure 2S. Comparison of the sex-specific risk ratios for age for all-cause and COVID-19 deaths in 16 countries. Risk ratios and 95% confidence intervals for the age (per 10 years) of COVID-19 mortality (*full circles*) and all-cause mortality (*empty circles*) in females (*red*) and males (*blue*) per country obtained from *Model 1 COVID* and *Model 1 ALL*.

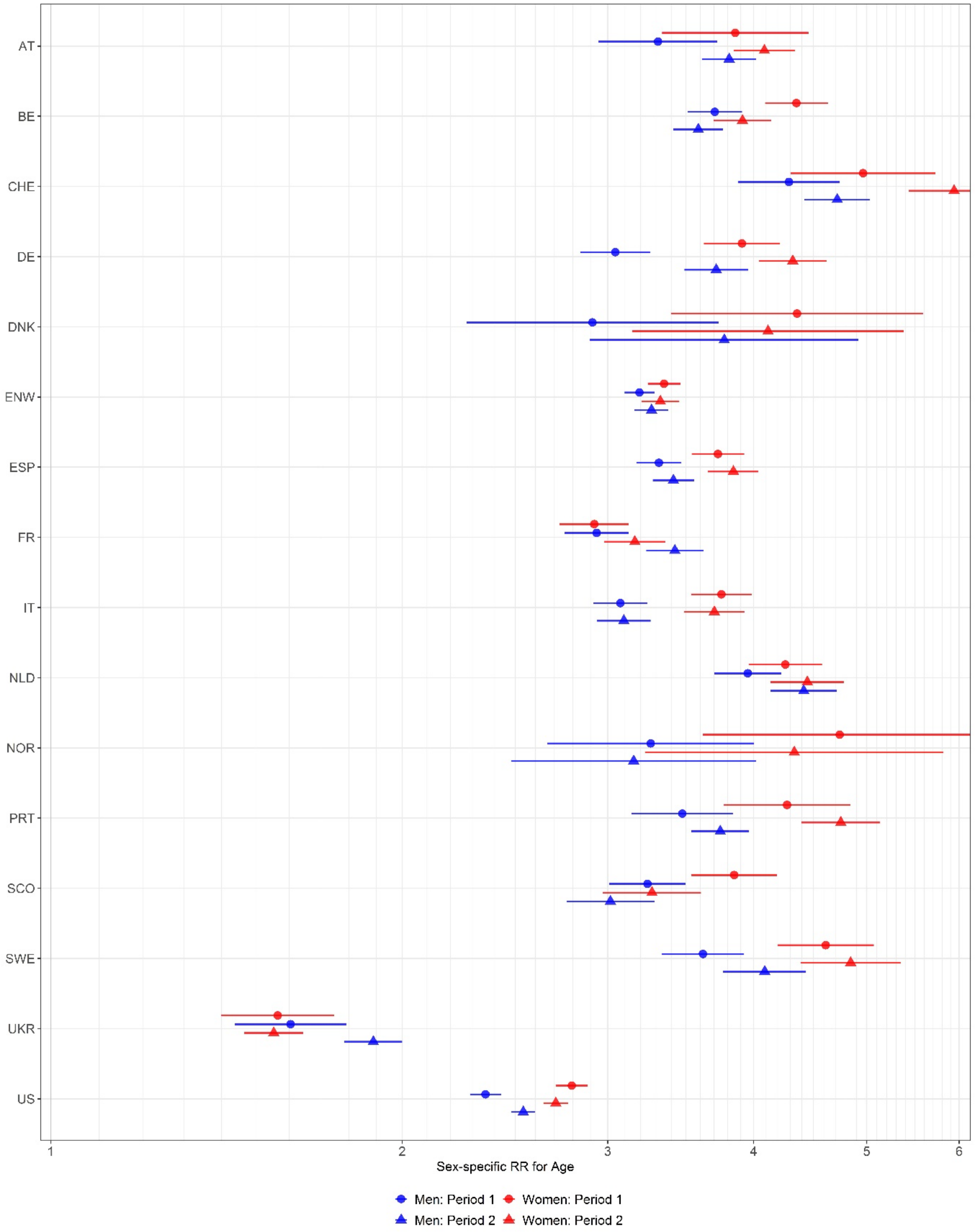

Figure 3S. Comparison of the sex- and period-specific risk ratios for the age-dependency of COVID-19 deaths in 16 countries. Sex-specific risk ratios and 95% confidence intervals for age (per 10 years) of COVID-19 mortality separately for the first (*full circles*) and second period (*full triangles*) in females (*red*) and males (*blue*) per country were obtained from *Model 2 COVID*.

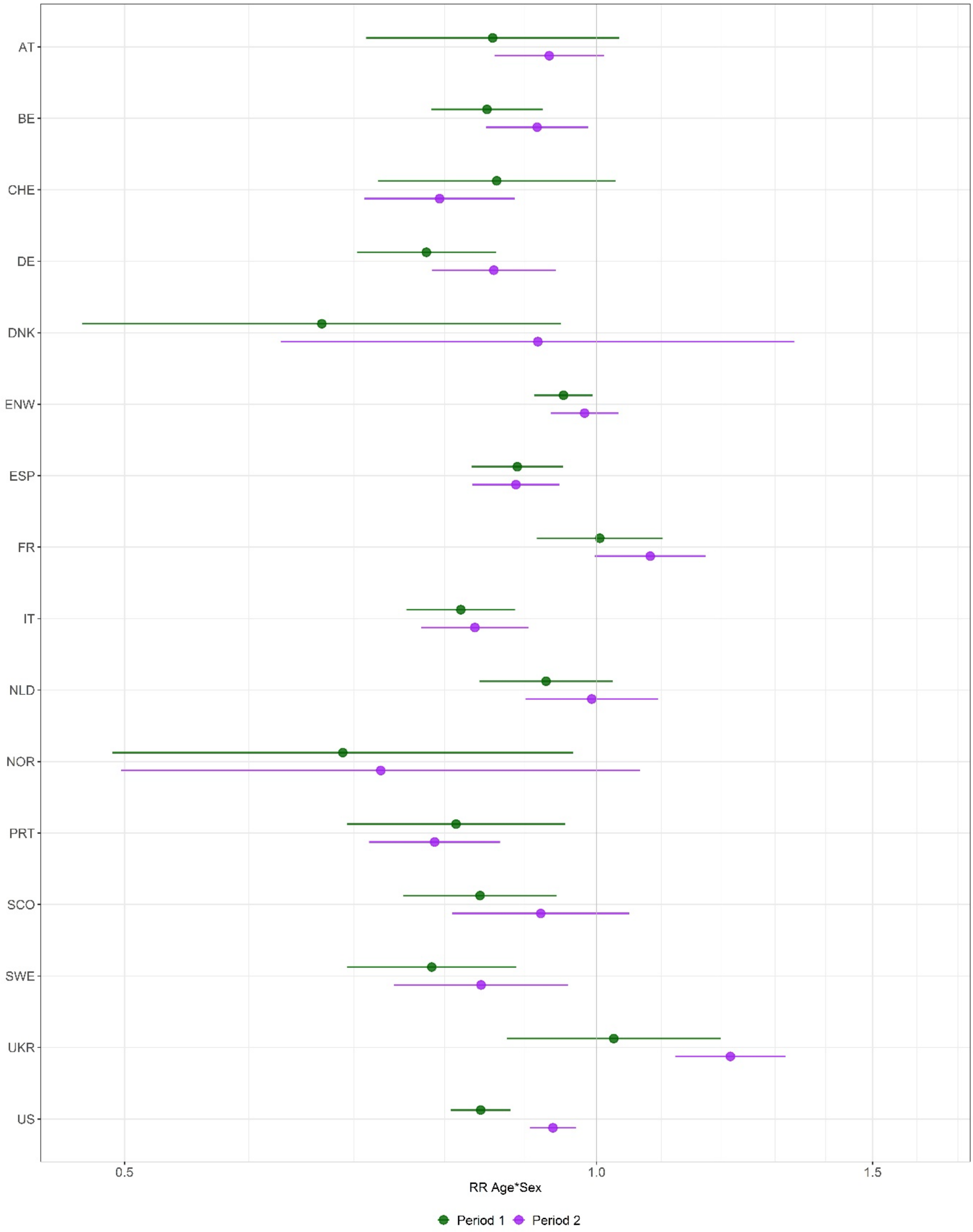

Figure 4S. Comparison of the interaction of sex × age between periods 1 and 2. Period-specific risk ratios and 95% confidence intervals for the interaction of age × sex (age per 10 years) of COVID-19 deaths separately for the first (*green*) and second period (*purple*) per country obtained from *Model 2 COVID*.
